# Traumatic brain injury: An EEG point of view

**DOI:** 10.1590/1980-57642016dn11-010002

**Published:** 2017

**Authors:** Jéssica Natuline Ianof, Renato Anghinah

**Affiliations:** 1 Department of Neurology, University of São Paulo Medical School, São Paulo SP – Brazil.

**Keywords:** EEG, quantitative EEG, traumatic brain injury, mild traumatic brain injury

## Abstract

Traumatic brain injury (TBI) is a silent epidemic. Mild traumatic brain injury (mTBI) causes brain injury that results in electrophysiologic abnormalities visible on electroencephalography (EEG) recordings. The purpose of this brief review was to discuss the importance of EEG findings in traumatic brain injury. Relevant articles published during the 1996-2016 period were retrieved from Medline (PubMed). The keywords were in English and included "traumatic brain injury", "EEG" and "quantitative EEG". We found 460 articles, analyzed 52 and selected 13 articles. EEG after TBI shows slowing of the posterior dominant rhythm and increased diffuse theta slowing, which may revert to normal within hours or may clear more slowly over many weeks. There are no clear EEG or quantitative EEG (qEEG) features unique to mild traumatic brain injury. Although the literature indicates the promise of qEEG in reaching a diagnosis and indicating prognosis of mTBI, further study is needed to corroborate and refine these methods.

## Abstract (pt)

O trauma cranioencefálico (TCE) é uma epidemia silenciosa. O trauma cranioencefálico leve causa uma lesão cerebral que resulta em anormalidades eletrofisiológicas visíveis nos registros eletroencefalográficos (EEG). O intuito dessa revisão curta foi discutir a importância dos achados eletroencefalográficos no trauma cranioencefálico. Os artigos relevantes publicados durante o período 1996-2016 foram selecionados do Medline (PubMed). As palavras-chave estavam em inglês e incluíam "*traumatic brain injury*", "EEG" e "quantitative EEG". Foram encontrados 460 artigos, analisados 52 e selecionados 13 artigos. O EEG após o TCE mostra lentificação do ritmo posterior dominante e aumento difuso da atividade lenta teta, o que pode ser revertido dentro de horas ou semanas. Não há características únicas no EEG ou EEG quantitativo (EEGq) de pacientes com TCE leve. Apesar da literatura indicar que no futuro o EEGq será uma ferramenta para diagnosticar e estabelecer um prognóstico para o TCE, mais estudos são necessários para corroborar e refinar esses métodos.

Traumatic brain injury (TBI) is an insult to the brain from an external mechanical force, which can lead to permanent or temporary impairment of cognitive, physical, and psychosocial functions.^1^

TBI is considered a "silent epidemic" because of its high incidence, great potential for disability and impact on the economy.^2^

Evidence of impaired brain function may include one or more of the following^3^:

- Loss of consciousness (LOC) or alteration of consciousness – the individual feels "disoriented" or "confused";
- Posttraumatic amnesia (PTA) - impairment of new learning immediately following the event, and, in some cases, events immediately preceding it;
- Focal neurological signs - motor, sensory, or reflex abnormalities, aphasia or dysphasia, or seizures (focal or generalized);
- Abnormalities on formal neuropsychological testing;
- Intracranial lesion(s) consistent with neurotrauma on computed tomography (CT) or magnetic resonance imaging (MRI) of the brain.

Electroencephalography (EEG) was the first clinical neurodiagnostic assessment that revealed abnormal brain function following traumatic brain injury.^4-6^

To detect brain injury, EEG may be more sensitive than clinical neurological examination. After mTBI, most patients (86%) with an abnormal neurological examination had an abnormal EEG. On the contrary, only 23% of abnormal EEGs were accompanied by an abnormal neurological examination^7^. EEG changes are not uniform across all individuals, due to differences in the severity of head injury. Some people have a clinically normal EEG as early as 15 minutes after concussion^8^.

EEG abnormalities are more commonly seen in patients with durations of unconsciousness lasting more than 2 minutes (56%) than in patients with briefer periods of unconsciousness (17%).^9^

The aim of this review was to discuss the importance of EEG findings in traumatic brain injury.

Relevant articles published during the 1996-2016 period were retrieved from Medline (PubMed). The keywords were in English and included " traumatic brain injury", "EEG" and "quantitative EEG". Studies were excluded if they addressed subjects with psychiatric disorders or involved children or young people. We found 460 articles. The initial selection of the articles was based on analysis of the abstracts and was performed independently by two researchers – we analyzed 52 and selected 13 articles for this brief review. Decisions concerning the inclusion or exclusion of articles were made jointly by the researchers.

**Electroencephalogram.** Conventional EEG refers to standard clinical (analog or, more commonly, digital) recording of electrical activity generated by the brain, as detected by scalp electrodes, presented as raw tracings of electrical waveforms and inspected visually by a qualified electroencephalographer. Higher density or other non-standard electrode arrays are sometimes used clinically but are more commonly applied to research recordings.^3^ Digital recording combined with software-assisted data analysis allows quantitative EEG interpretation (qEEG) and identification of subtle shifts in the types and patterns of EEG activity.^3^ The most commonly used qEEG measures are:

- Spectral analysis - frequency composition of the EEG over a given period;
- Absolute and relative amplitude (µV/cycle/second) and power (µV^2^/cycle/second) within a frequency range or at each channel;
- Coherence;
- Symmetry between homologous pairs of electrodes.

It is important to note that there are no clear EEG or qEEG features unique to mild traumatic brain injury.

**Acute EEG changes after mild traumatic brain injury (mTBI) .** Immediately after mTBI, there is epileptiform activity (high amplitude sharp waves or high frequency discharges), followed by diffuse suppression of cortical activity typically lasting 1–2 min, and followed by diffuse slowing of the EEG, which returns to the normal baseline within 10 min to 1 h.^10-12^ qEEG most commonly shows immediate reduction in mean alpha frequency,^13^ with increased theta,^14,15^ increased delta,^16^ or increased theta:alpha ratio.^17,18^

**Subacute EEG changes in mTBI.** Weeks to months after mTBI there is a 1–2 Hz increase in the frequency of the posterior alpha rhythm, which has been explained as a return to the original baseline from the post-traumatic slowing.^7,19^ The majority of the acute EEG abnormalities described above resolve by 3 months, and 90% resolve within 1 year of the head trauma.^19^

**Chronic EEG changes in mTBI.** Lewine et al. (2007) studied a group of 30 patients with persistent (>1 year) psychiatric, somatic, or cognitive complaints developing within the first few weeks of mTBI. The magnetoencephalography (MEG) revealed epileptiform abnormalities in 16% and slow-wave abnormalities in 63%.^20^

A higher power in the delta band (1.5–5 Hz) and a lower power in the alpha band (8.5–12 Hz) were seen in postconcussive syndrome patients compared with matched controls.

## CONCLUSION

Conventional EEG is important for the evaluation of posttraumatic epilepsy but is not useful as a routine screening measure among individuals with mTBI or postconcussive symptoms. Quantitative EEG appears promising as a diagnostic assessment for mTBI and postconcussive symptoms. Further scientific studies are needed to provide a better understanding of the pathophysiology and elucidate how EEG can assist in the care of patients who have sustained an mTBI.
